# Structure, Folding Dynamics, and Amyloidogenesis of D76N β_2_-Microglobulin: ROLES OF SHEAR FLOW, HYDROPHOBIC SURFACES, AND α-CRYSTALLIN*

**DOI:** 10.1074/jbc.M113.498857

**Published:** 2013-09-06

**Authors:** P. Patrizia Mangione, Gennaro Esposito, Annalisa Relini, Sara Raimondi, Riccardo Porcari, Sofia Giorgetti, Alessandra Corazza, Federico Fogolari, Amanda Penco, Yuji Goto, Young-Ho Lee, Hisashi Yagi, Ciro Cecconi, Mohsin M. Naqvi, Julian D. Gillmore, Philip N. Hawkins, Fabrizio Chiti, Ranieri Rolandi, Graham W. Taylor, Mark B. Pepys, Monica Stoppini, Vittorio Bellotti

**Affiliations:** From the ‡ Wolfson Drug Discovery Unit, Centre for Amyloidosis and Acute Phase Proteins, Division of Medicine, University College London, London NW3 2PF, United Kingdom,; the § Department of Molecular Medicine, Institute of Biochemistry, University of Pavia, 27100 Pavia, Italy,; the ¶ Department of Medical and Biological Sciences, University of Udine, 33100 Udine, Italy,; the ‖ Department of Physics, University of Genoa, 16146 Genoa, Italy,; the ** Institute for Protein Research, Osaka University, Osaka 565-0871, Japan,; the ‡‡ Consiglio Nazionale delle Ricerche-Nanoscience Institute S3, 41125 Modena, Italy,; the §§ Department of Physics, University of Modena and Reggio Emilia, 41125 Modena, Italy, and; ¶¶ Department of Biochemical Sciences, University of Florence, 50134 Florence, Italy

**Keywords:** Amyloid, Protein Aggregation, Protein Misfolding, Protein Stability, Shear Stress, D76N β_2_-Microglobulin, Chaperones, Systemic Amyloidosis

## Abstract

Systemic amyloidosis is a fatal disease caused by misfolding of native globular proteins, which then aggregate extracellularly as insoluble fibrils, damaging the structure and function of affected organs. The formation of amyloid fibrils *in vivo* is poorly understood. We recently identified the first naturally occurring structural variant, D76N, of human β_2_-microglobulin (β_2_m), the ubiquitous light chain of class I major histocompatibility antigens, as the amyloid fibril protein in a family with a new phenotype of late onset fatal hereditary systemic amyloidosis. Here we show that, uniquely, D76N β_2_m readily forms amyloid fibrils *in vitro* under physiological extracellular conditions. The globular native fold transition to the fibrillar state is primed by exposure to a hydrophobic-hydrophilic interface under physiological intensity shear flow. Wild type β_2_m is recruited by the variant into amyloid fibrils *in vitro* but is absent from amyloid deposited *in vivo*. This may be because, as we show here, such recruitment is inhibited by chaperone activity. Our results suggest general mechanistic principles of *in vivo* amyloid fibrillogenesis by globular proteins, a previously obscure process. Elucidation of this crucial causative event in clinical amyloidosis should also help to explain the hitherto mysterious timing and location of amyloid deposition.

## Teaser

**Background:** We recently discovered the first natural human β_2_-microglobulin variant, D76N, as an amyloidogenic protein.

**Results:** Fluid flow on hydrophobic surfaces triggers its amyloid fibrillogenesis. The α-crystallin chaperone inhibits variant-mediated co-aggregation of wild type β_2_-microglobulin.

**Conclusion:** These mechanisms likely reflect *in vivo* amyloidogenesis by globular proteins in general.

**Significance:** Our results elucidate the molecular pathophysiology of amyloid deposition.

## Introduction

β_2_m^2^ (mass, 11,729 Da), the invariant light chain of the human HLA class I complex, is produced at ∼200 mg/day in adults and is cleared only via the kidney. In patients with end stage renal failure on dialysis, the plasma concentration of β_2_m therefore rises from the normal 1–2 mg/liter to persistently raised values of ∼ 50–70 mg/liter, leading to the serious and intractable condition of dialysis-related amyloidosis with β_2_m amyloid fibrils deposited in bones and joints, causing painful arthropathy, bone cysts, pathological fractures, and rarely visceral β_2_m amyloid deposits. The normal structure and function of β_2_m are well characterized, and although wild type β_2_m is poorly amyloidogenic *in vitro*, its fibrillogenesis and its tissue-specific deposition have been intensively investigated (1). Despite much progress, there is neither general agreement about the underlying molecular mechanisms nor an understanding of the forces involved *in vivo* during the destabilization and subsequent amyloid aggregation of either β_2_m or any of the other natively folded globular proteins that form amyloid fibrils in disease. We lately reported (2) the first naturally occurring structural variant of β_2_m, D76N, discovered in members of a French family who developed progressive bowel dysfunction with extensive visceral β_2_m amyloid deposits despite normal renal function and normal circulating β_2_m concentrations and with none of the osteoarticular deposits characteristic of dialysis-related amyloidosis. Here we elucidate in detail the biophysical parameters of amyloid fibrillogenesis by this uniquely tractable protein and develop a paradigm that could be applicable generally to the *in vivo* pathophysiology of amyloidogenesis by the whole range of globular proteins that cause clinically significant systemic amyloidosis.

## EXPERIMENTAL PROCEDURES

### 

#### 

##### Production of Recombinant Proteins

Recombinant wild type and variant β_2_m were expressed and purified as described previously (2).

##### Differential Scanning Calorimetry

Differential scanning calorimetry experiments were carried out with a VP-DSC instrument (MicroCal, Northampton, MA) with protein at 0.5 mg/ml in 25 mm sodium phosphate buffer, pH 7.4 and scans from 10 to 90 °C at a scanning rate of 60 °C/h. The reversibility of protein denaturation was assessed by repeating heating and cooling cycles. After normalization and base-line subtraction, the thermal unfolding curves were analyzed using MicroCal Origin 7.0 software with a two-state unfolding model.

##### Equilibrium Denaturation Experiments and Folding Kinetics

Guanidine hydrochloride (Gdn-HCl) equilibrium denaturation, unfolding, and refolding kinetics were performed as described previously (3). All experiments were carried out at 30 °C in 20 mm sodium phosphate buffer, pH 7.4 at a 0.02 mg/ml final protein concentration. Refolding of acid-denatured protein and double jump experiments were performed at 4 °C as described previously (4).

##### Energy Diagram

All free energy changes (Δ*G*) were determined in J mol^−1^ and then converted into kcal mol^−1^; throughout we use the following abbreviations: U, unfolded state; I, intermediate; N, native state; subscript C, *cis*-His^31^-Pro^32^; subscript T, *trans*-His^31^*-*Pro^32^; TS, transition state. The U_T_ state was arbitrarily given a free energy (*G*) of 0 J mol^−1^ and was considered as a reference for all reported Δ*G* values. The Δ*G* from the U_T_ to the N_C_ states was determined from Gdn-HCl unfolding equilibrium curves as reported (3). The Δ*G* from the N_T_ to the N_C_ states was determined using Δ*G* = −*RT*ln(*k*_u_/*k*_slow_) where *R* is the universal gas constant, *T* is the absolute temperature, and *k*_u_ and *k*_slow_ are the rate constants (in s^−1^ units) for unfolding and for the slow phase of folding, respectively, extrapolated to the absence of Gdn-HCl. The Δ*G* from the I_T_ to the U_T_ states was determined by plotting the fluorescence of the I_T_ state (corresponding to the fluorescence at time 0 of a kinetic trace of folding) against Gdn-HCl concentration and by plotting the fluorescence of the U_T_ state against Gdn-HCl concentration (in the latter case, the values at low Gdn-HCl concentration were obtained by linear extrapolation from the values at high Gdn-HCl concentration). The fluorescence of the I_T_ state decreased with increasing Gdn-HCl concentration until it approached the fluorescence of the U_T_ state, thus providing an approximate measure of the conformational stability of the I_T_ state relative to U_T_. The Δ*G* from the I_T_ to the TS_2_ state was determined using Δ*G*^‡^ = −*RT*ln(*k*_fast_/*k*^‡^) where Δ*G*^‡^ is the free energy barrier, *k*_fast_ is the rate constant for the fast phase of folding, and *k*^‡^ is the pre-exponential term taken as 4.8 10^8^ s^−1^ as reported (5, 6). Similarly, the Δ*G* values from N_T_ to TS_3_ and from N_C_ to TS_3_ were determined using Δ*G*^‡^ = −*RT*ln(*k*_slow_/*k*^‡^) and Δ*G*^‡^ = −*RT*ln(*k*_u_/*k*^‡^), respectively, where *k*_slow_ and *k*_u_ are the rate constants for the slow phase of folding and for unfolding, respectively. The Δ*G* values from U_T_ to TS_1_ and from I_T_ to TS_1_ were not determined. All other Δ*G* values not explicitly mentioned in the study can be determined by arithmetic linear combination of the Δ*G* parameters described above.

##### NMR Measurements

NMR spectra were obtained at 500.13 MHz with a Bruker Avance 500 spectrometer on 0.1–0.3 mm protein samples dissolved in H_2_O/D_2_O 90:10 or 95:5 with 20–70 mm sodium phosphate buffer and pH* (pH meter reading without isotope effect correction) in the range 6.6–7.2. Unlabeled and uniformly ^15^N- or ^15^N,^13^C-labeled protein samples, expressed as described previously (2), were used. The spectra were collected mostly at 25 °C with only a few experiments obtained also at 30 or 37 °C. Homonuclear two-dimensional TOCSY (7), NOESY (8), and ROESY (9) spectra were acquired. The adopted experimental schemes included solvent suppression by WATERGATE (10) and excitation sculpting (11); 1-s steady state recovery time; mixing times (*t_m_*) of 40–50 ms for TOCSY, 100–150 ms for NOESY, and 100 ms for ROESY; *t*1 quadrature detection by time-proportional phase incrementation (12); and gradient-assisted coherence selection (echo-antiecho) (13). The spin-lock mixing of the TOCSY and ROESY experiments was obtained with MLEV17 (14) pulse trains or single long pulse, respectively, at γ*B*_2_/2π ∼10 kHz (TOCSY) and ∼ 2.5 or 5 kHz (ROESY). The acquisitions were performed over a spectral width of 8012.82 Hz in both dimensions with matrix size of 1024–2048 points in *t*2 and 256–400 points in *t*1 and 32–64 scans per each *t*1 free induction decay. The BLUU-Tramp experiments were conducted using the procedure described previously over the temperature range 22–42 °C (15, 16). Measurements were performed on samples that had undergone complete deuterium substitution for hydrogen with two cycles of exchange at 4 °C in D_2_O containing 10 mm NH_4_HCO_3_ and subsequent lyophilization. The solvent for the back-exchange was used for the preliminary shimming to enable quick start after dissolving the protein (∼5 min dead time before starting the acquisition). The ^15^N{^1^H} NOE data were obtained at 25 and 37 °C by standard sequence using a 3-s relaxation interval. The spectra with (NOE) and without (no NOE) proton saturation were acquired in an interleaved manner.

Three-dimensional HNCA (17–19) and HNCOCA (19, 20) were typically acquired with 64 scans and 64 × 40 × 1024 data points in *t*1 (^13^C), *t*2 (^15^N), and *t*3 (^1^H), respectively, over spectral windows of 40, 33.5, and 16 ppm for ^13^C, ^15^N, and ^1^H, respectively. HNCO spectra (17, 19) were acquired using 128 × 40 × 1500 data points and only 32 scans for each *t*1 × *t*2 experiment over spectral windows of 22.1 (^13^C), 33.5 (^15^N), and 14 (^1^H) ppm. Processing of three-dimensional data ended up with real matrices of 512 × 256 × 1024 points in F1, F2, and F3, respectively, except for the HNCO spectra where the carbon dimension (F1) was limited to 256 points. All data, except those from BLUU-Tramp, were processed with Topspin (Bruker Biospin) and analyzed with Sparky (T. D. Goddard and D. G. Kneller, University of California). BLUU-Tramp data were processed using NMRPipe and analyzed by NMRView (21).

##### Electrostatic Calculations

For the calculation of both surface potential and p*K_a_* shifts, we used the recently developed program BLUUES (22) available also as a server utility (23). For the calculation of isopotential surfaces, we used the program UHBD, and we displayed the isopotential surfaces using the program VMD. To assess effects that could arise from slightly different arrangement in the structural models used for calculation, an alternative structure for the D76N variant was generated using the program SCWRL4.0 by alternative schemes: (i) only the side chain of the mutated residue is allowed to change conformation, and (ii) only the side chain of the mutated residues and contacting residues are allowed to change conformation. Despite numerical differences, the results from the homology-modeled structures are in agreement with the experimental data reported in the study, confirming that the effects are mainly due to the mutation rather than other minor conformational differences.

##### Molecular Dynamics Simulations

The force field used in the simulations was CHARMM v.27 (24) with the CMAP (two-dimensional dihedral energy grid correction map approach) correction (25). The minimized system was further relaxed, keeping the solute molecules (including ions) fixed, by molecular dynamics simulation. The system was heated to 47 °C in 2 ps, and a further 18-ps simulation was run to let water molecules reorient, consistent with the average lifetime of a hydrogen bond in water of 1–2 ps (26). The system without restraints on solute molecules was energy-minimized by 300 conjugate gradient minimization steps. The system was then heated to 47 °C in 2 ps, and a further 3.0-ns simulation was run to reach equilibrium. The simulations lasted 250 ns, and snapshots were saved every 0.1 ns. All molecular dynamics simulations were performed in the NPT ensemble using the Nosé-Hoover Langevin piston method (27, 28). The Langevin damping coefficient for temperature control was 10 ps^−1^. For all simulations, the size of the box was fluctuating around its average value within fractions of Å.

##### H-bond Network Analysis

The mutation D76N within the β_2_m sequence is likely to affect the molecular hydrogen bond network. To identify indirect effects of the mutation, 2500 snapshots (at 100-ps intervals) were taken from molecular dynamics simulations, and hydrogen bonds were listed using the program MOLMOL (29). Only hydrogen bonds involving at least one side chain group were selected to remove nonspecific effects. Each hydrogen bond was taken as representative of the proximity of the two involved residues, *r*_1_ and *r*_2_. In particular, a distance, *d_r_*_1,_*_r_*_2_, was assigned based on the ratio between the number of snapshots where the hydrogen bond is present (*n_s_*) and the total number of snapshots (*n*_tot_).

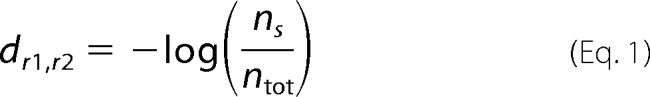


Once a set of pairwise distances had been assigned in this way, the shortest path between all nodes was found using the Floyd-Warshall algorithm, which outputs the path and the nodes along the path. In this way, the shortest (in the sense of most frequently observed) network of hydrogen bonds connecting different residues was identified. This definition has the obvious disadvantage of not considering whether hydrogen bonds are observed simultaneously or not. On the other hand, it takes advantage of being based only on pairwise connection, enabling the applicability of the fast Floyd-Warshall algorithm. The output of the program readily identifies stable or fluctuating networks of hydrogen bonds.

##### Fibrillogenesis

Fibrillogenesis experiments were performed in standard quartz cells stirred at 1500 rpm and 37 °C using 40 μm β_2_m isoforms in 25 mm sodium phosphate, pH 7.4 containing 10 μm thioflavin T (ThT). Aggregation was carried out without seeds of preformed fibrils. ThT emission was monitored at 480 nm after excitation at 445 nm using a PerkinElmer Life Sciences LS 55 spectrofluorometer. Fibrillogenesis experiments were also conducted without agitation or in the absence of the air-water interface and with addition of 6 μm elastin isolated from human aorta (Sigma-Aldrich). β_2_m, which remained soluble during fibrillogenesis experiments, was monitored by native gel electrophoresis (30). The soluble fractions were separated by centrifugation at 17,000 × *g* for 15 min before loading onto 1% agarose gel, and bands were quantified with Quantity One software (Bio-Rad). The effects of 1 and 40 μm α-crystallin (Sigma-Aldrich) on fibrillogenesis by an equimolar mixture of 40 μm wild type and D76N β_2_m, respectively, were investigated, and soluble fractions of the two isoforms were quantified at 24 h by native agarose gel electrophoresis as described above.

##### Atomic Force Microscopy

Tapping mode AFM images were acquired in air using a Dimension 3000 scanning probe microscope equipped with a “G” scanning head (maximum scan size, 100 μm) and driven by a Nanoscope IIIa controller and a Multimode scanning probe microscope equipped with an “E” scanning head (maximum scan size, 10 μm) and driven by a Nanoscope V controller (Digital Instruments, Bruker). Single beam uncoated silicon cantilevers (type OMCL-AC160TS, Olympus) were used. The drive frequency was between 320 and 340 kHz; the scan rate was 0.5–2.0 Hz. All aggregation experiments were carried out with a 0.4 mg/ml protein concentration in 25 mm sodium phosphate, pH 7.4 at 37 °C with stirring at 750 rpm.

##### Aggregation Time Course by AFM

Aliquots (2 μl) were withdrawn at time 0, 1, 2, 8, and 24 h, respectively. After 500-fold dilution, 10 μl were finally deposited on freshly cleaved mica and dried under mild vacuum.

##### Effect of Graphite Sheets by AFM

Aggregation was conducted with and without agitation. Aliquots (2 μl) of samples under agitation were diluted 100-fold, and 10 μl were deposited on freshly cleaved mica and dried under mild vacuum. A graphite sheet was removed from the non-stirred protein solution, gently rinsed with purified (Milli-Q) water, fixed on a metallic disk, and dried under mild vacuum.

##### Ultrasonication

The effect of carbon nanotubes (0.01 mg/ml) on D76N β_2_m fibrillogenesis was evaluated by a combination of a water bath type ultrasonicator and microplate reader (HANABI, Elekon Science Co. Ltd. and Corona Electric Co., Japan). Protein at 40 μm in 25 mm sodium phosphate, pH 7.4, 10 μm ThT with or without nanotubes was aliquoted in a 96-well plate. Cycles of ultrasonication with simultaneous shaking (3 min) followed by 7-min quiescence were sequentially applied to the plate. The frequency and output of the sonication were set to 20 kHz and 700 watts, respectively, and the temperature was kept at 37 °C. Emission of ThT fluorescence was recorded at the end of ultrasonication/shaking treatment.

##### Protein Adsorption at Hydrophobic Surfaces

The structure of a protein adsorbed at a hydrophobic-hydrophilic interface is perturbed by interfacial, intermolecular, and hydrophobic interactions (31–34). However, studies suggest that among these destabilizing interactions, hydrophobic forces play a dominant role (35–37). Many authors have shown that the interaction of a protein with an apolar surface can be triggered by exposed hydrophobic domains (37–40). If we consider the exposed hydrophobic domains present on β_2_m, we can estimate the forces acting on the protein once it is absorbed on an apolar surface using a model recently proposed to calculate hydrophobic interaction energies. According to this model, the hydrophobic interaction energies (*E*_hydro_) between two apolar surfaces can be described by the following model (41).

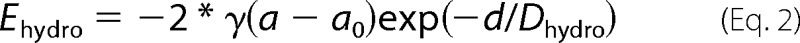
 where γ is the interfacial tension, *d* is the distance between the two surfaces, *a* is the exposed area of the molecule at distance *d*, *a*_0_ is the optimum exposed area of the molecule, which we consider to be equal to the area of one amino acid, and *D*_hydro_ is the hydrophobic decay length. Thus, the hydrophobic force (*F*_hydro_) acting on the molecule can be calculated as follows.

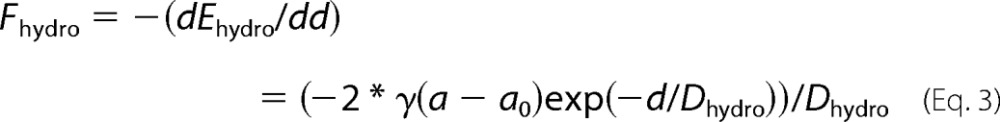


For our system, we can consider that γ = 50 mJ/m^2^ (42), *a_0_* = 50 Å^2^, *D*_hydro_ = 10 Å (43), and *a*(*d*) = *a*_0_(1 − exp(−*d*/*D*_hydro_))^−1/2^. Using these values, it can be calculated that when the distance between the protein and the surface ranges between 1 and 10 Å the interaction energies and forces acting on the molecule vary from 14.7 to 0.7 kcal mol^−1^ and from 4.8 to 102 piconewtons, respectively. These forces/energies should be large enough to perturb the three-dimensional structure of β_2_m molecules (2, 44, 45).

## RESULTS

### 

#### 

##### Structural Basis of Amyloidogenicity of D76N β_2_m Variant

The Asp^76^ mutation to Asn substantially destabilizes β_2_m, and differential scanning calorimetry reveals a melting temperature 10.26 °C lower than that of the wild type, corresponding to mean ± S.D. (*n* = 3) Δ*H* values of 63.9 ± 1.2 and 86.2 ± 1.5 kcal mol^−1^ for D76N and wild type β_2_m, respectively. The calculated Δ*G* values (46) for unfolding of the D76N variant at 37 and at 30 °C were thus 2.73 and 2.86 kcal mol^−1^ lower, respectively, than for wild type β_2_m.

**FIGURE 1.**
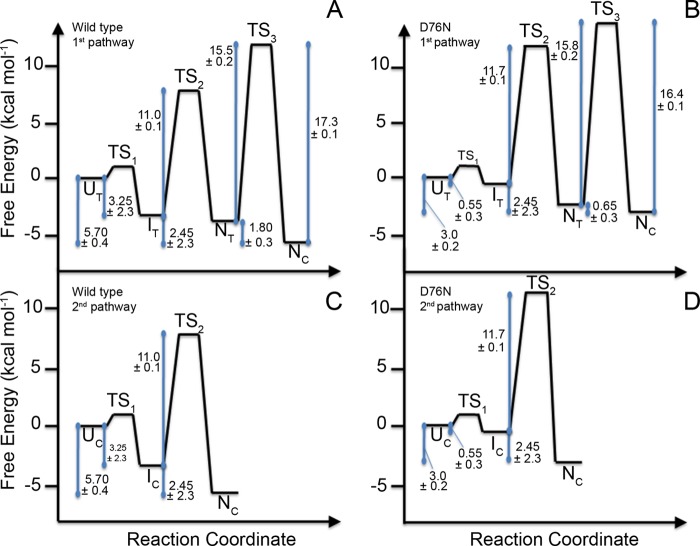
**Free energy diagram for wild type and variant β_2_m on the two parallel pathways from the unfolded to the native state.** *A* and *B*, free energy values related to the slower pathways limited by the *trans* → *cis* isomerization of the His^31^-Pro^32^ bond. *C* and *D*, free energy values associated with the rapid formation of native-like molecules via an intermediate containing the native isomer *cis*-His^31^-Pro^32^ (I_C_).

The complex folding mechanisms shared by wild type and D76N β_2_m involve multiple intermediates and parallel folding routes with two major exponential phases observed during refolding (3): an initial fast phase that was 3 times slower for the D76N variant at 0.2 m Gdn-HCl and a subsequent prolyl *trans-cis* isomerization-dependent slow phase (4) (data not shown). Unresolved fluorescence changes take place in the dead time of the experiments, showing that a burst phase occurs on the submillisecond time scale with the same amplitude for both proteins. In contrast to folding, unfolding appears to be a monophasic process. D76N β_2_m unfolded faster than wild type with a 2-fold increase at 5.4 m Gdn-HCl. The free energy diagrams of (un)folding of the two proteins at pH 7.4, 30 °C in the absence of any denaturant (Fig. 1) derived from combined equilibrium unfolding and kinetic data (Table 1) show that the N_C_ native state of the variant is (mean ± S.D.; *n* = 3) 2.7 ± 0.25 kcal mol^−1^ less stable than that of native wild type β_2_m, thereby promoting the population of partially folded, typically amyloidogenic states of the variant (47). The native-like state of β_2_m with the His^31^-Pro^32^ peptide bond in a non-native *trans* configuration (N_T_), previously shown to be highly related to the amyloidogenic pathway of wild type β_2_m and populated at (mean ± S.D.; *n* = 3) 4.8 ± 3.0% at equilibrium, was remarkably more abundant in the D76N variant (mean ± S.D.; *n* = 3) at ∼25 ± 9%.

**TABLE 1:**
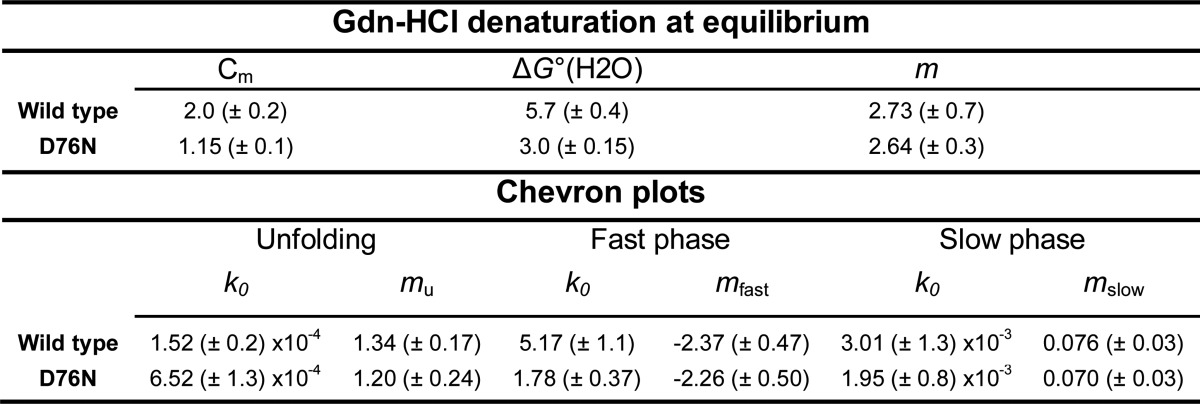
**Thermodynamic and kinetic values** All values are mean ± S.D. (*n* = 3) · *C_m_* (m), midpoint concentration of Gdn-HCl; Δ*G*^0^(H_2_O) (kcal mol^−1^), free energy of unfolding in the absence of denaturant; *m* (kcal mol^−1^ m^−1^), dependence of Δ*G*^0^(H_2_O) on denaturant concentration; *k*_0_ (s^−1^), value of rate constant extrapolated to zero denaturant concentration. These values were used to calculate the free energy changes shown in Fig. 1. In addition, Δ*G* values from the I_T_ to the U_T_ states, 3.25 ± 2.3 and 0.55 ± 0.3 kcal mol^−1^ for wild type and D76N β_2_m, respectively, were determined with the procedure described under “Experimental Procedures.” *m*_u_, *m*_fast_, and *m*_slow_ (kcal mol^−1^ m^−1^), dependence of ln*k* on Gdn-HCl concentration for unfolding and fast and slow phases of folding, respectively.

**FIGURE 2.**
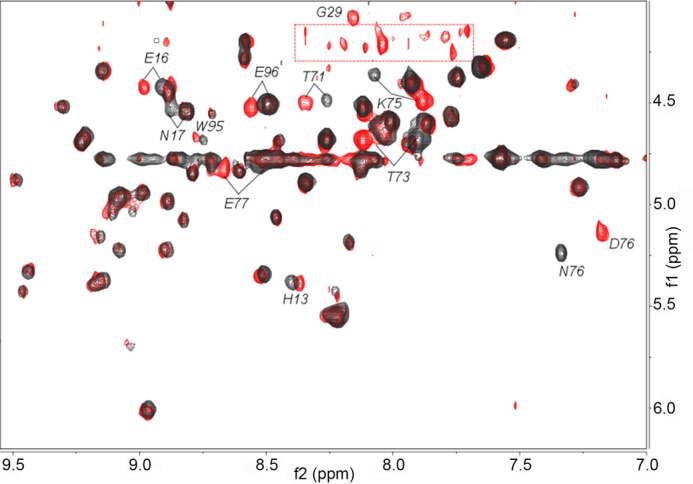
**Overlay of the 500-MHz TOCSY fingerprints.** Spectra of D76N (*black* contours) and wild type β_2_m (*red* contours) were monitored at 25 °C. The different cross-peaks of the mutation site, *i.e.* Asp^76^ and Asn^76^, are indicated. Other differences are observed for the H^N^-H^α^ connectivities of residues Lys^75^ and Glu^77^ flanking the mutation site, although changes also occur at the close Thr^71^ and Thr^73^ residues. Additional shifts can be detected for the backbone amides of His^13^, Glu^16^, Asn^17^, Trp^95^, and Glu^96^ that are located in the same apical region as the loop including the mutation site and are likely to depend on local solvation changes. The *boxed* region in the spectrum of the wild type protein encloses resonances from a limited population of non-natively folded structures arising from the partial unfolding occurring in aged samples. The Gly^29^ H^N^-H^α^ cross-peak is undetectable for the variant protein because of fast exchange under the experimental conditions.

Despite the notably reduced stability of D76N β_2_m, its solution structure did not differ significantly from wild type. Other than obvious changes at the mutation site and neighboring residues, the NMR signature of the variant at 25 °C was nearly the same as that of the wild type protein (Fig. 2).

**FIGURE 3.**
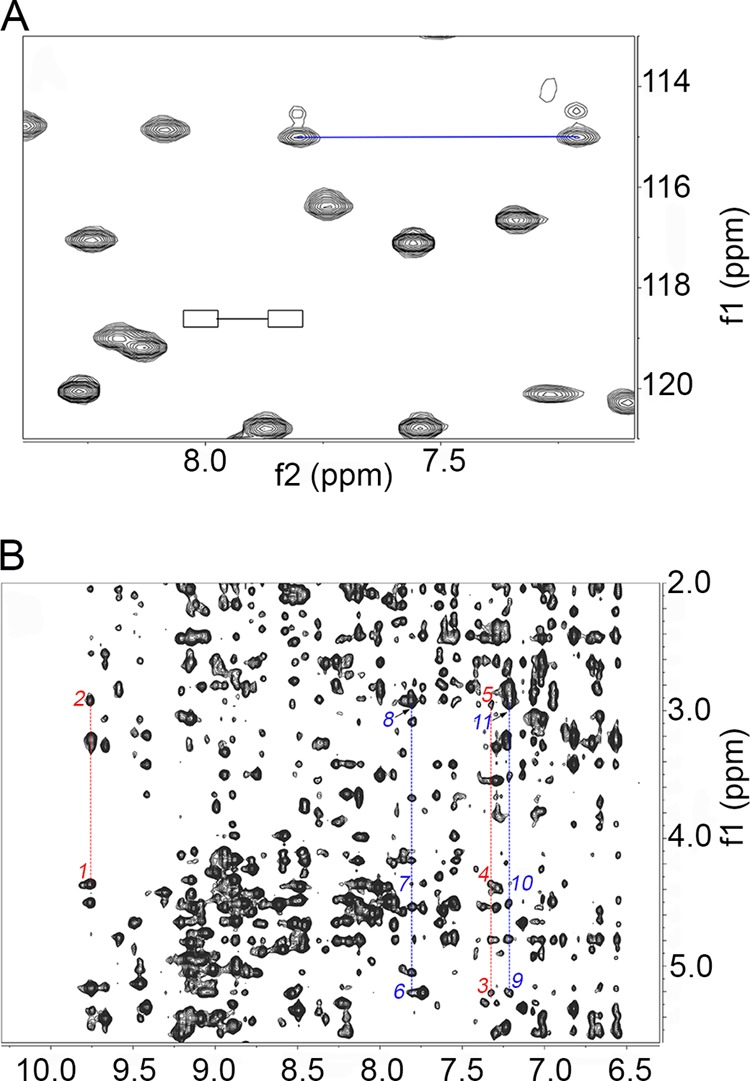
**NMR patterns of Asn^42^ and Asn^76^ carboxyamides.** *A*, region of ^15^N-^1^H heteronuclear single quantum correlation spectrum of D76N β_2_m. The *boxed* extremes of the *black line* correspond to the locations expected for the carboxyamide resonances of Asn^42^ in the wild type protein spectrum; the extremes of the *blue line* indicate the new signals observed for D76N β_2_m. The new signal pair can be attributed to Asn^76^ and Asn^42^ side chain amides from NOESY evidence. *B*, details from ^1^H two-dimensional NOESY spectrum of D76N β_2_m. The *dashed red lines* cross through the backbone amide NOEs of Asn^42^ (9.75 ppm) and Asn^76^ (7.31 ppm), whereas the *dashed blue lines* cross through the NOEs of the carboxyamide NHs at 7.80 and 7.21 ppm that are attributed to Asn^42^ and Asn^76^ and tentatively assigned as H^δ21^ and H^δ22^, respectively. To avoid notation crowding, the relevant connectivities are highlighted by *numbers* (*1*, Asn^42^ NH-Asn^42^ H^α^; *2*, Asn^42^ NH-Asn^42^ H^β^; *3*, Asn^76^ NH-Asn^76^ H^α^; *4*, Asn^76^ NH-Asn^42^ H^α^; *5*, Asn^76^ NH-Asn^76^ H^β^; *6*, Asn^76^/Asn^42^ H^δ21^-Asn^76^ H^α^; *7*, Asn^76^/Asn^42^ H^δ21^-Asn^42^ H^α^; *8*, Asn^76^/Asn^42^ H^δ21^-Asn^76^/Asn^42^ H^β^; *9*, Asn^76^/Asn^42^ H^δ22^-Asn^76^ H^α^; *10*, Asn^76^/Asn^42^ H^δ22^-Asn^42^ H^α^; *11*, Asn^76^/Asn^42^ H^δ21^-Asn^76^/Asn^42^ H^β^, with the slash indicating the lack in stereospecific assignment). The NOESY cross-peaks observed for the backbone amides of Asn^76^ and Asn^42^ as well as for the carboxyamide resonances at 7.21 and 7.80 ppm support the attribution of the latter signal pair to the carboxyamides of the same residues. Observing the same chemical shifts for the carboxyamide resonances of both Asn^76^ and Asn^42^ suggests that the two side chains experience the same environment, which in turn is consistent with the occurrence of reciprocal H-bonds between Asn^42^ and Asn^76^ carboxyamides.

Unequivocal assignment at 11.7 teslas (500-MHz ^1^H frequency) could be obtained for 85% of the backbone ^1^H, ^15^N, and ^13^C nuclei, but no major chemical shift difference, that is no major structure deviation, was observed compared with the wild type protein, consistent with the crystallographic findings (2). For instance, the average difference in deviation from random coil values of the H^α^ chemical shifts between D76N and wild type β_2_m, Δ(Δδ), is −0.012 ± 0.017 ppm with a value of −0.01 ppm even at the mutation site. This reflects essentially invariant local secondary and tertiary structure. One relevant effect related to the mutation is that the same chemical shifts are observed for the carboxyamide resonances of both Asn^42^ and Asn^76^ (Fig. 3*A*), suggesting that the two side chains share the same chemical environment, consistent with the occurrence of reciprocal H-bonds between the carboxyamides. The presence of an interaction between the side chains of residues 42 and 76 in the variant protein is supported by several lines of evidence including two-dimensional ^1^H NOESY (Fig. 3*B*) and ROESY cross-peaks (data not shown).

**FIGURE 4.**
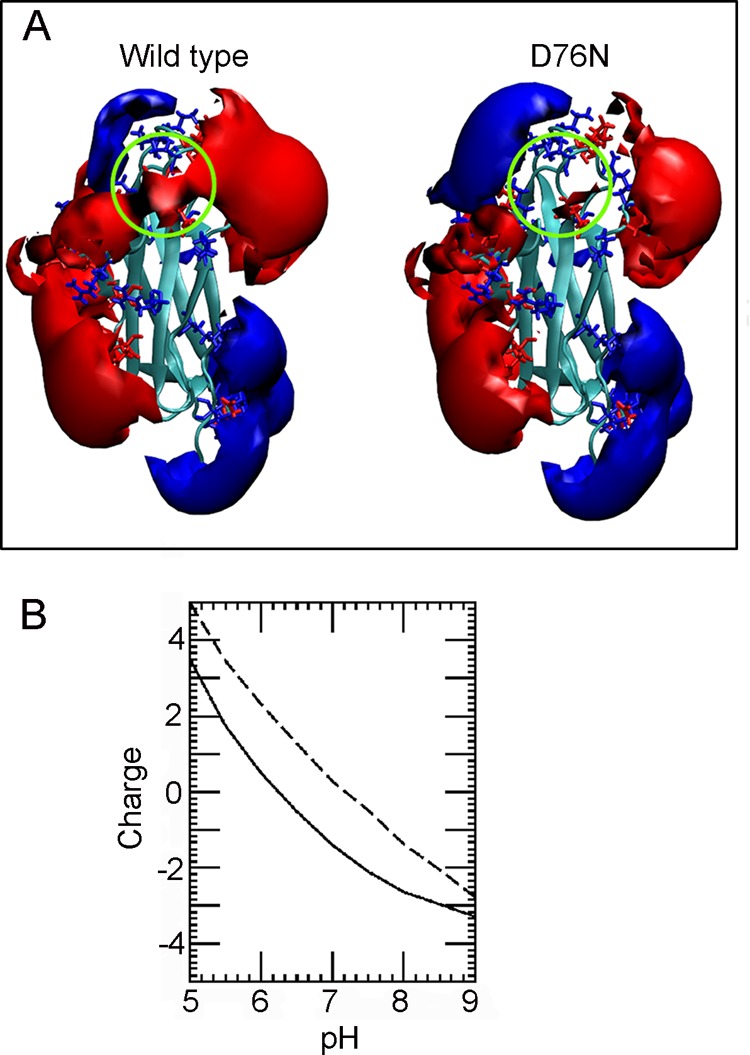
**Electrostatic properties of wild type and D76N β_2_m.** *A*, isopotential curves are displayed at +0.5*kT*/*q* (*blue*) and −0.5*kT*/*q* (*red*). The regions around Asp^76^ and Asn^76^ are *circled*. The molecular structure of β_2_m (Protein Data Bank code 3HLA, chain B) (74) and the D76N β_2_m homology model were prepared for electrostatic calculations using the program PDB2PQR, which adds hydrogens and assigns charges and atomic radii according to different force fields. The CHARMM force field parameters were used except that the minimum radius was set at 1.0 Å. Similar results are obtained from the crystallographic structure of the variant (Protein Data Bank code 4FXL) (2). *B*, relationship between total net charge (elementary charge units) and pH for wild type (*solid line*) and D76N β_2_m (*dashed line*). Total charge was calculated by summing the charges of all ionizable groups for which individual p*K_a_* values had been calculated using BLUUES (22).

**FIGURE 5.**
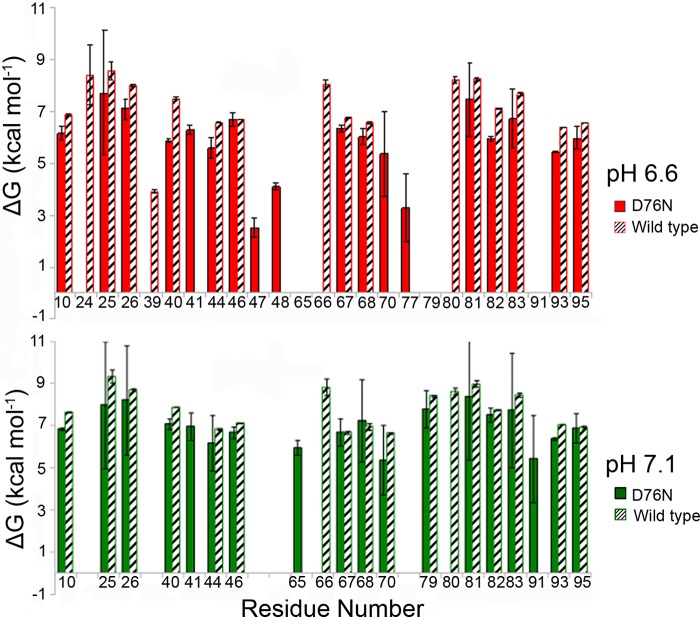
**BLUU-Tramp analysis.** Δ*G*_unfol_ values according to residue number from the amide exchange-based approach BLUU-Tramp (15, 16) are shown as histograms for D76N and wild type β_2_m at different pH values. Despite the instability of the variant during the experiment, a Δ*G*_unfol_ difference of 1.8 ± 1.2 kcal mol^−1^ at pH 7.1, 30 °C was assessed between wild type and variant β_2_m. This value is consistent, within the experimental error, with the destabilization (2.86 kcal mol^−1^) estimated more precisely by calorimetry under similar conditions. *Error bars* represent S.E. Both species show similar patterns of structural determinants, that is residues exhibiting the largest opening Δ*G* values and that presumably represent the global opening process. Note that these residues were selected among the resolved resonances exhibiting an observable isotope exchange pattern within the designated temperature interval. Larger *error bars* for some residues of the variant compared with wild type are due to experimental error in the quantification of the cross-peak of the unstable variant. Nonetheless, in addition to reduction of Δ*G*_unfol_, further destabilization was evident in residues 40–44 of the variant (C strand end, CC′ loop). Destabilization of the weaker connection between loops EF and CC′ should also affect the hydrophobic packing of residues 39–40 and 79–80 with an instability propagation path linked to the F strand bearing the disulfide bridge to the B strand, which is the core of the immunoglobulin domain architecture.

In the wild type protein, the side chain amide of Asn^42^ is H-bonded to the Asp^76^ side chain carboxylate that also forms salt bridges with the side chain ammoniums of Lys^41^ and Lys^75^. The latter salt bridges contribute electrostatic stability and a computed p*K_a_* shift of −1.2 units for the Asp^76^ carboxylate with respect to the standard value (Table 2). Despite the survival of the residue 42-76 interaction, the asparagine substitution for aspartate has a substantial impact in the variant protein. At pH 7.0, the net charge of D76N β_2_m is +0.3 units, whereas wild type carries an average −1.4 elementary charge (Fig. 4). This is consistent with a decreased intermolecular repulsion that facilitates aggregation at pH around neutrality. In addition, the lower stability of D76N β_2_m compared with wild type as shown by microcalorimetry was confirmed at single residue resolution by NMR using BLUU-Tramp (15, 16) even under critical conditions for the variant due to its instability (Fig. 5).

**TABLE 2:** **pK_a_ values for groups titrating below pH 10 in wild type and D76N β_2_m** Values were calculated with the software BLUUES (22) and compared with the corresponding random coil model values (limit). The p*K_a_* values for Lys^41^ side chain, which establishes an electrostatic interaction with Asp^76^ in wild type β_2_m, are also given. Asp^76^ is the lowest titrating residue with a p*K_a_* of 2.6, corresponding to a p*K_a_* shift of 1.2 compared with the free amino acid p*K_a_* of 3.8. This shift is primarily due to the interaction with Lys^41^ and Lys^75^. Among other groups with a significant shift of p*K_a_* are Asp^34^ (more exposed to the solvent than Asp^53^) and Asp^38^ (similar degree of exposure limitation and interactions as Asp^76^). Among histidine residues, His^31^ and His^84^ both have a p*K_a_* shifted toward acidic pH by ∼2 p*K* units.

Residue	p*K_a_*(limit)	p*K_a_* WT β_2_m	p*K_a_*D76N β_2_m
Ile^1^	8.000	7.388	7.401
His^13^	6.500	6.078	6.006
Glu^16^	4.500	3.679	3.668
His^31^	6.500	4.641	4.630
Asp^34^	3.800	3.117	3.127
Glu^36^	4.500	4.211	4.207
Asp^38^	3.800	3.492	3.495
Glu^44^	4.500	4.678	4.599
Glu^47^	4.500	4.119	4.120
Glu^50^	4.500	4.927	4.940
His^51^	6.500	6.967	6.956
Asp^53^	3.800	3.564	3.589
Asp^59^	3.800	3.727	3.711
Glu^69^	4.500	4.579	4.535
Glu^74^	4.500	4.191	4.100
Asp^76^	3.800	2.619	
Glu^77^	4.500	4.056	4.089
His^84^	6.500	4.471	4.525
Asp^96^	3.800	5.350	5.338
Asp^98^	3.800	4.374	4.408
Met^99^	3.200	4.863	4.836
Lys^41^	10.500	11.173	10.470

**FIGURE 6.**
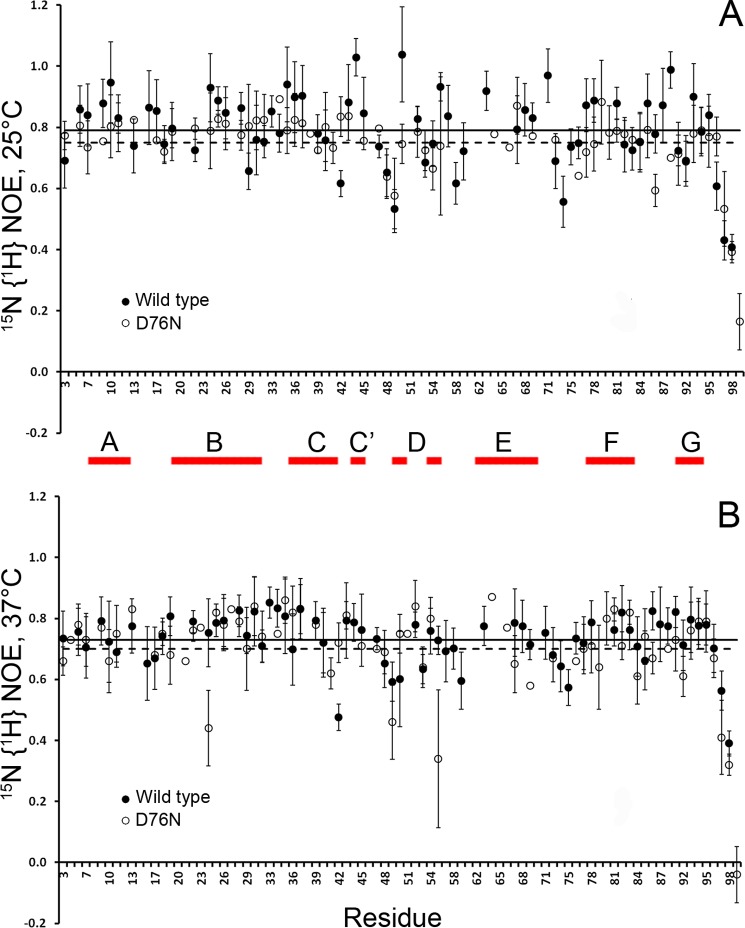
**Temperature and heteronuclear NOE.** ^15^N{^1^H} NOE values measured at 11.7 teslas (500.13-MHz ^1^H frequency) at 25 (*A*) and 37 °C (*B*) for wild type and D76N β_2_m. The *horizontal lines* represent the average values for wild type (*solid line*) and variant (*dashed line*) species; *error bars* indicate S.E. The *abscissa axes* do not include the positions 5, 14, 32, 72, and 90 corresponding to proline residues, therefore lacking in secondary amides. Only the pairs of well resolved cross-peaks observed at both temperatures were selected for each species. The strand naming scheme is drawn *parallel* to *abscissas* between the two panels.

**FIGURE 7.**
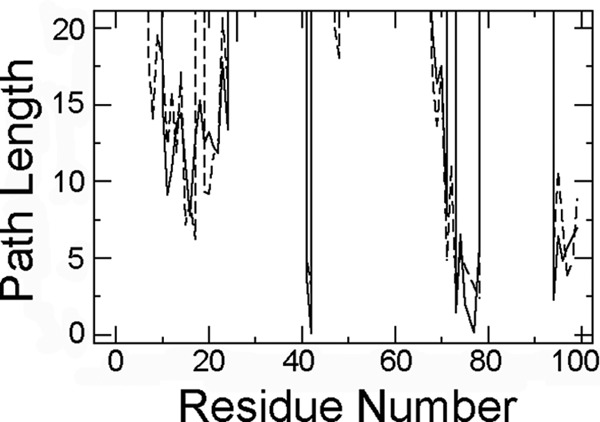
**Hydrogen bonding relationship of the residue 76 side chain.** Shortest hydrogen bond path lengths involving at least one side chain atom for residue 76 in wild type (*solid line*) and D76N (*dashed line*) β_2_m from the Floyd-Warshall algorithm are shown. The variant shows only minor differences from wild type, specifically with a slightly stronger connection of residue 76 to loop AB and overall slightly weaker connection to other residues of the loop EF itself, to loop CC′D, and to the C terminus. The wild type Asp^76^ residue is strongly linked to Asn^42^ with the hydrogen bond Asn^42^ H^δ2^-Asp^76^ O^δ1^, which is found in 2222 of 2500 snapshots. In turn, Asn^42^ is hydrogen-bonded with Glu^77^ via two nearly completely conserved hydrogen bonds (Asn^42^ H^N^-Glu^77^ O and Glu^77^ H^N^-Asn^42^ O^δ1^). The connection to the C*-*terminal residue Lys^94^ is due to the salt bridge with Glu^77^. The connection with the loop AB is weaker and involves the hydrogen bonds of Asp^76^ with Thr^73^ (Thr^73^ H^γ1^-Asp^76^ O^δ1^), the fluctuating hydrogen bond of Arg^97^ side chain with Thr^73^ backbone, and the salt bridge of Arg^97^ with Glu^16^. The connection between Thr^73^ and Arg^97^ is very weak. In D76N β_2_m, all connections are weaker except for the path to loop AB with a hydrogen bond of the side chain of Arg^97^ to the side chain of Asn^76^ and the hydrogen bond of the side chain of Arg^97^ to the side chain of Asn^17^.

**FIGURE 8.**
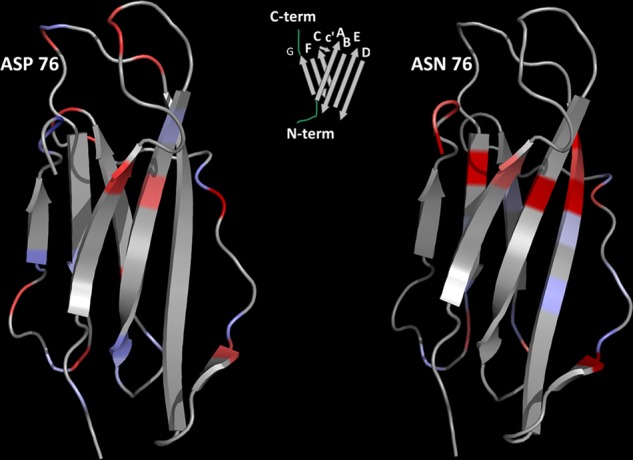
**Changes in backbone mobility.** The mobility of wild type and D76N β_2_m based on the heteronuclear NOE ratios derived from values shown in Fig. 6 with increments (*red* intensity) and decrements (*blue* intensity) is shown. The weaker connection between EF and CC′ loops of the variant spreads all around the underlying region with flexibility gains involving the intervening edges of strands A, B, E, and F. The solution structure of β_2_m (Protein Data Bank code 1JNJ) was also used to model the D76N variant. The *central* diagram shows the strand naming scheme.

The lower thermal resistance of D76N β_2_m can be explicitly tracked from ^15^N{^1^H} NOE data measured at 25 and 37 °C, respectively (Fig. 6). These data indicate an extended loss of rigidity consistent with the H-bond analysis (Fig. 7). The average NOE values at 25 °C are consistent with a higher mobility of the variant compared with the wild type. The loss of rigidity observed when temperature increases at 37 °C is slightly more marked for the wild type protein (Fig. 6). However, the fluctuations of the thermally induced mobility increment that derive from the NOE_25__°C_/NOE_37__°C_ ratio are uniformly spread over the whole molecule of the wild type protein compared with a distinctive uneven pattern in the variant. When related to the spatial structure, this pattern delineates an instability propagation path (illustrated by the backbone mobility changes with temperature shown in Fig. 8). Decreased conformational stability and reduction of repulsive electrostatic interactions make D76N β_2_m extremely sensitive to aqueous boundary conditions where the preferential interface partitioning of the protein and the subsequent surface tension fluctuations overcome the determining role for the effective force field of hydrophobic folding drives, thereby enhancing unfolding and fibrillogenic nucleation events.

##### Fibrillogenesis of D76N β_2_m Occurs under Physiological Conditions

**FIGURE 9.**
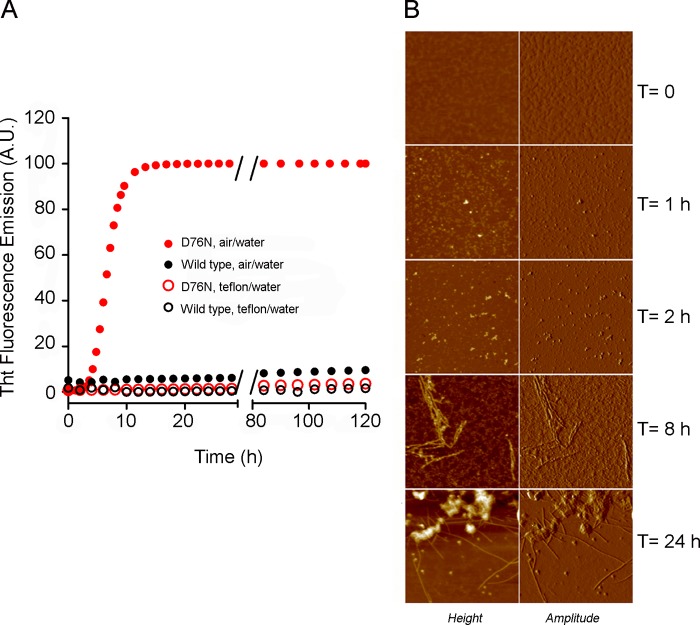
**Fibrillogenesis of D76N and wild type β_2_m.** *A*, the time course of aggregation of D76N β_2_m (*red*) and wild type (*black*) was monitored under stirring conditions by fluorescence emission of ThT (using 445 and 480 nm as excitation and emission wavelengths, respectively). Proteins were dissolved at 40 μm in 25 mm sodium phosphate buffer, pH 7.4 at 37 °C. Aggregation experiments were monitored in the presence of air-water (*filled circles*) and Teflon-water interfaces (*empty circles*). *B*, tapping mode AFM images of different stages of aggregation of D76N β_2_m carried out under stirring conditions and in the presence of an air-water interface. Oligomers formed after 1 h, prefibrillar aggregates coexisted with oligomers after 2 h, filaments were observed after 8 h, and fibril clusters were observed after 24 h. Scan size, 1 μm; Z range, 15 (times 0 and 24 h), 8 (times 1 and 2 h), and 3 nm (time 8 h). *a.u.*, arbitrary units.

In marked contrast to the wild type protein, D76N β_2_m rapidly aggregates as shown by ThT fluorescence (Fig. 9*A*) and atomic force microscopy (Fig. 9*B*) when agitated at pH 7.4 and 37 °C in the presence of an air-water interface. We had already shown that neither protein aggregates in the absence of agitation (2). Furthermore, replacement of the air-water interface with Teflon-water, which reduces the interfacial tension from about 70 to 50 millinewtons/m (43) at fixed interfacial area, also completely suppressed aggregation of the D76N variant (Fig. 9*A*).

**FIGURE 10.**
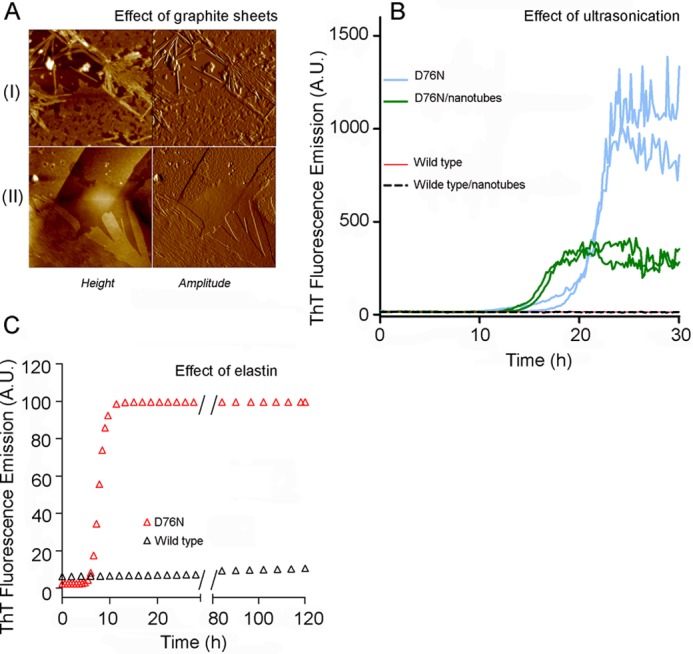
**Fibrillogenesis of D76N and wild type β_2_m in presence of hydrophilic-hydrophobic interfaces.** *A*, tapping mode AFM images of fibrils formed by D76N β_2_m in the presence of graphite sheets under stirring conditions (*I*) or without agitation (*II*). Scan size, 2 μm, Z range, 15 (*I*) and 25 nm (*II*). *B*, time course of fibril formation by D76N β_2_m under ultrasonication (*light blue lines* show replicate experiments) in contrast to the absence of fibrillogenesis by wild type β_2_m (*red line*) under the same conditions. D76N β_2_m fibril formation was accelerated in the presence of carbon nanotubes (*green lines*), whereas wild type β_2_m (*dashed black line*) did not aggregate. *C*, fibrillogenesis of D76N (*red triangles*) and wild type β_2_m (*black triangles*) carried out under stirring conditions at 37 °C in the presence of a Teflon-water interface with 6 μm human elastin. *a.u.*, arbitrary units.

The water-air boundary is known to behave as a hydrophobic interface (43, 48, 49). To evaluate interfacial effects on D76N β_2_m fibrillogenesis, the air-water interface was removed and replaced with a hydrophilic-hydrophobic interface. We used the prototypic hydrophobic surface provided by graphite or elastin, the very hydrophobic ubiquitous insoluble fibrillar component of the extracellular matrix (50) (Fig. 10). In the absence of the air boundary, graphite triggered fibril formation on the sheet surfaces (Fig. 10*A*) without massive conversion of the bulk β_2_m in solution (data not shown). Ultrasonication triggered fibrillogenesis by D76N β_2_m even in the absence of an air-water interface (Fig. 10*B*, *blue lines*), and aggregation was accelerated by addition of carbon nanotubes (Fig. 10*B*, *green lines*). In these conditions, ultrasonication had no effect on aggregation of wild type β_2_m (*red* and *dashed black lines*). Inclusion of fibrillar elastin in the protein solution, kept at 37 °C under agitation in the absence of an air boundary, strongly promoted fibril formation by D76N β_2_m (Fig. 10*C*, *red triangles*). Elastin did not promote aggregation by wild type β_2_m under the same conditions (Fig. 10*C*, *black triangles*).

Under conditions that suppress aggregation, the absence of an air-water interface or any agitation of the protein solution, the consistent enhancement of D76N β_2_m fibrillogenesis by graphite or elastin clearly demonstrates the impact of an increased interfacial area. In addition to the crucial role of hydrophobic-hydrophilic interfaces, shaking of the solution is required for amyloid conversion of D76N β_2_m in the bulk, and the mechanism by which agitation influences the kinetics of fibril formation is clearly important.

##### Role of Shear Forces and Hydrophobic Surfaces in β_2_m Amyloidogenesis

Agitation of a protein solution applies hydrodynamic shear stress, which in principle could also contribute to protein destabilization leading to denaturation (51). We have therefore calculated the shear forces acting on the β_2_m molecule using the equation (52)

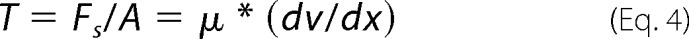
 where *T* is the shear stress, *F_s_* is the shear force, *A* is the cross-sectional area of the molecule, μ is the dynamic viscosity of the fluid, and *dv*/*dx* is the shear rate, that is the fluid velocity gradient. *F_s_* clearly depends greatly on molecular shape (for example, see Refs. 51 and 53). With uniform fluid flow, the major forces acting on a molecule in the bulk are elongational forces along the flow axis that depend on both protein length and shape. We have used the model proposed by Shankaran and Neelamegham (54) that assumes that the molecule has a dumbbell shape, which yields a force coefficient derived from the radius of the two ends of the dumbbell and the distance between them. *F_s_* is then calculated from

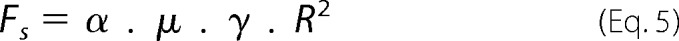
 where α is the force coefficient, μ is the dynamic viscosity, γ is the shear rate (*dv*/*dx*), and *R* is the radius of the molecule. The shear rate of the flow can be calculated as

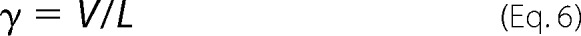
 where *V* is the translational velocity of the fluid and *L* is the half-length of the cell. For our system (Fig. 9*A*), γ = 94.2/s, and using an α value of 10 as reported previously for a similar system (54), the calculated shear force, *F_s_*, of 3.3 × 10^−17^ newton is much lower than the force of ∼10^−12^ newton typically required to unfold proteins (44, 45). Shear stress alone is thus unlikely to destabilize native β_2_m, but liquid agitation increases the sampling frequency of natively folded monomers at the hydrophobic-hydrophilic interface and facilitates the exchange of misfolded monomers and interface-formed nuclei of aggregation with the bulk solution. All these entities are then available for the recruitment of other protein units, thereby increasing the efficiency of aggregation (51).

At the hydrophobic-hydrophilic interface, the native protein fold is perturbed by the combined action of interfacial, intermolecular, and hydrophobic interactions (31–34) of which the latter is apparently dominant (35–37) with much evidence that exposed hydrophobic domains trigger the interaction of a protein with an apolar surface (37–40). Based on the known exposed hydrophobic domains present on β_2_m, we estimate that the forces acting on the protein at the hydrophobic-hydrophilic interface are in the range of 5–100 piconewtons and are sufficient to perturb its three-dimensional structure (2, 44, 45) (see “Experimental Procedures” for details).

The crucial role of interfacial forces in protein destabilization and fibrillogenesis must vary with the different electrostatic charges and thermodynamic stabilities of individual proteins because efficient adsorption at a hydrophobic-hydrophilic interface depends on overcoming the energy barriers of surface pressure and electrostatic repulsion (39, 55–58). The almost neutral D76N β_2_m molecule is likely to adsorb more rapidly than the more charged wild type β_2_m, and once at the interface, it should undergo larger structural perturbations as it is thermodynamically less stable (59, 60) (Fig. 9*A*).

##### D76N β_2_m Primes the Fibrillar Conversion of Wild Type β_2_m in Vitro

**FIGURE 11.**
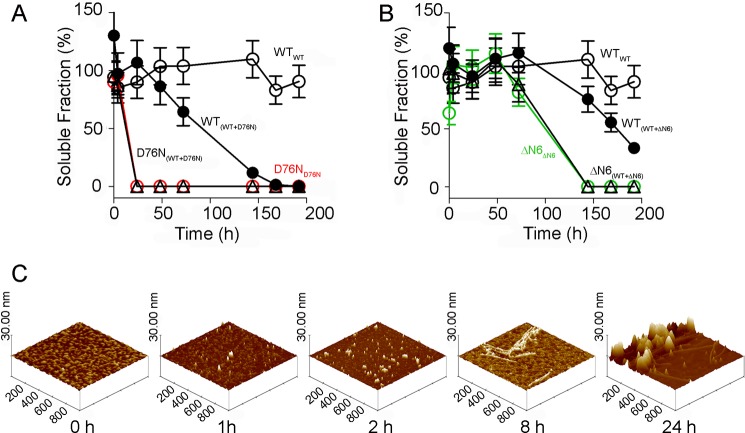
**Wild type β_2_m elongates D76N fibrils *in vitro*.** *A*, soluble fractions of wild type β_2_m either alone (*black empty circles*; *WT_WT_*) or in an equimolar mixture with the variant (*black filled circles*; *WT_(WT+D76N)_*) and of D76N variant either alone (*red empty circles*; *D76N_D76N_*) or in the mixture (*black empty triangles*; *D76N_(WT+D76N)_*). *B*, soluble fractions of wild type either alone (*black empty circles*; *WT_WT_*) or in an equimolar mixture with the variant (*black filled circles*; *WT*_*(WT*+Δ*N6)*_) and of ΔN6 β_2_m either alone (*green empty circles*; Δ*N6*_Δ*N6*_) or in the mixture (*black empty triangles*; Δ*N6*_*(WT*+Δ*N6)*_). Values are mean and S.D. (*error bars*) from three independent experiments. *C*, surface plots of AFM images showing different steps of the aggregation process of D76N β_2_m. At 1 h, oligomers are present; at 2 h, they coexist with short prefibrillar aggregates; and at 8 h, filaments can be observed, whereas at 24 h, fibrils and complex fibril assemblies are seen. The surface plots were obtained from topographic tapping mode AFM images (Fig. 9*B*).

**FIGURE 12.**
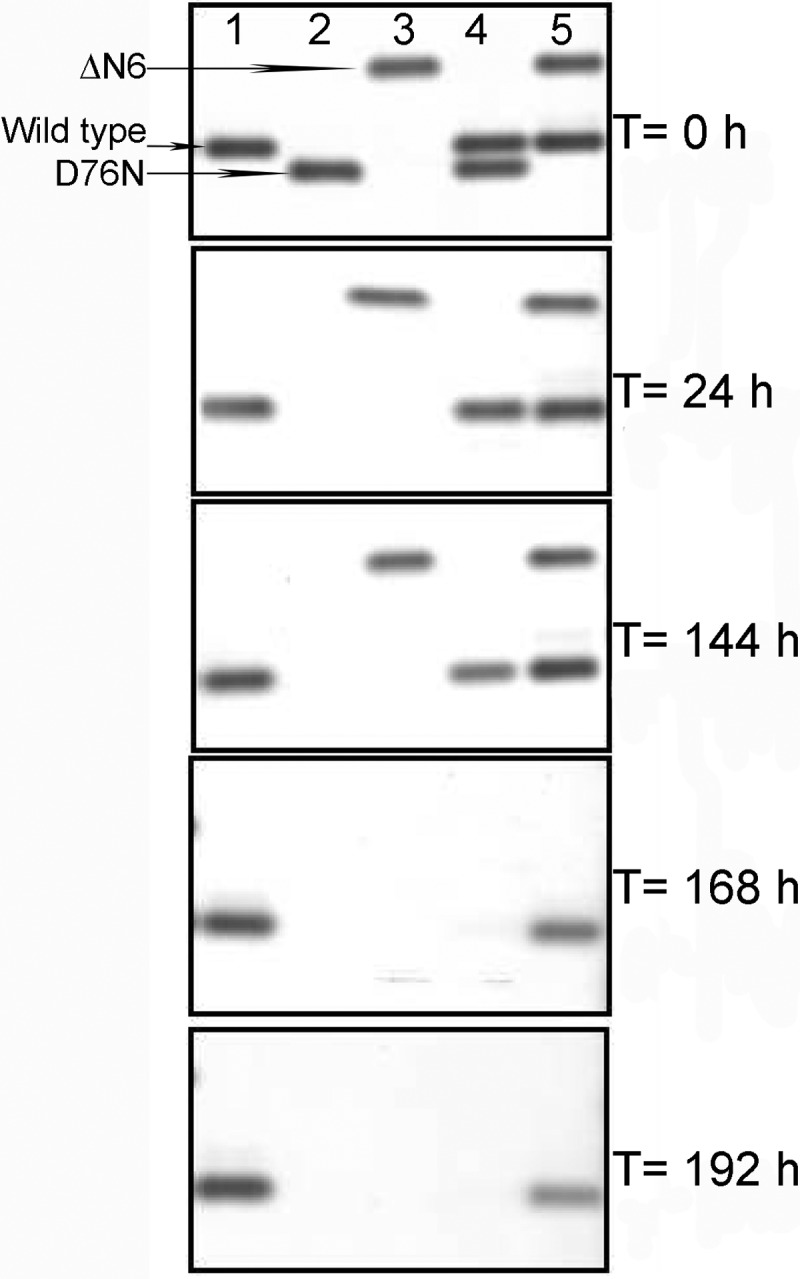
**Residual soluble β_2_m during aggregation.** Agarose gel electrophoresis analysis of supernatants from fibrillogenesis of wild type β_2_m alone (*lane 1*), D76N β_2_m alone (*lane 2*), ΔN6 β_2_m alone (*lane 3*), an equimolar mixture of wild type and D76N β_2_m (*lane 4*), and an equimolar mixture of wild type and ΔN6 β_2_m (*lane 5*) is shown. The *arrows* show the electrophoretic mobility of each isoform.

The D76N β_2_m variant in solution in physiological buffered saline converts into fibrils at the highest rate ever reported for an amyloidogenic globular protein under these conditions. When mixed in equimolar proportions with native wild type β_2_m, all the latter was also transformed into insoluble fibrils (Fig. 11*A*) with a much shorter lag phase than reported previously for seeding by the truncated isoform lacking the six N-terminal residues (ΔN6 β_2_m) (61) (Fig. 11*B*). Fibrillogenesis was monitored by quantifying the soluble protein by native 1% agarose gel electrophoresis in which the soluble forms of wild type, D76N, and ΔN6 β_2_m are readily distinguished by their different respective electrophoretic mobilities (Fig. 12). The duration of the lag phase depended on the aggregation state of D76N variant, which can potently promote aggregation of wild type β_2_m only when it is assembled into elongated oligomers and filaments (Fig. 11*C*).

**FIGURE 13.**
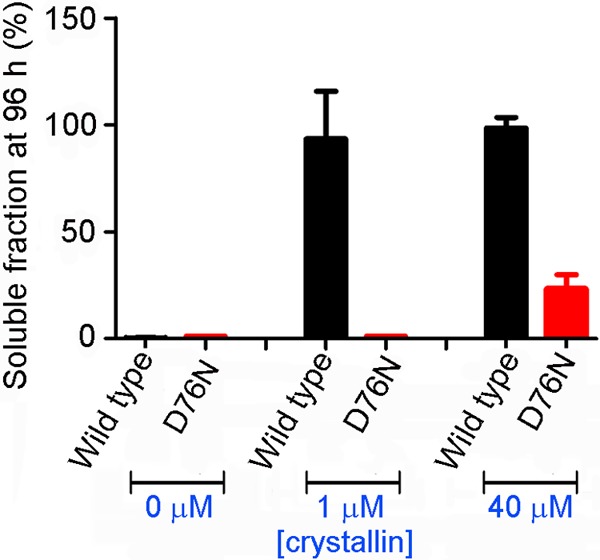
**Modulation by α-crystallin of fibril formation by wild type and D76N β_2_m.** Soluble fractions of wild type and D76N variant β_2_m from an equimolar mixture in the presence and absence of α-crystallin quantified at 96 h by native agarose gel electrophoresis are shown. Values are mean ± S.D. (*error bars*) from three independent experiments.

Unexpectedly, our previous proteomic characterization of *ex vivo* natural amyloid fibrils from the tissue deposits of patients carrying the amyloidogenic D76N mutation showed only the presence of full-length variant protein (2). Because wild type β_2_m is intrinsically amyloidogenic *in vivo* and forms abundant amyloid fibrils in patients affected by dialysis-related amyloidosis, the absence of any wild type β_2_m in the hereditary variant β_2_m deposits indicates that *in vivo* fibrillogenesis is more complex than the simple *in vitro* experiment containing just wild type and variant β_2_m. A likely physiological factor modulating misfolding, aggregation, and fibrillogenesis could be the presence of extracellular chaperones. Indeed, we show here that α-crystallin (62, 63) prevented amyloid conversion of wild type β_2_m induced by D76N β_2_m fibrils without interfering with fibrillogenesis of the variant at the lowest chaperone concentration used (1 μm). However, at 40 μm α-crystallin, even the conversion of D76N β_2_m is significantly reduced (Fig. 13). The effect of this prototypic chaperone strongly suggests mechanisms responsible for the observed composition of naturally occurring fibrils in the affected kindred (2).

## DISCUSSION

β_2_m is among the most extensively studied globular protein precursors of human amyloid fibrils. The discovery of the first natural variant of human β_2_m as the cause of hereditary systemic amyloidosis uniquely enables a very informative comparison of two different types of β_2_m amyloidosis with distinctly different clinical and pathological features. The D76N residue substitution allows a fully folded three-dimensional structure almost identical to that of the wild type protein that forms amyloid fibrils in dialysis-related amyloidosis. However, dissection of the mechanism of D76N β_2_m fibrillogenesis confirmed our previously established paradigm that the amyloidogenicity of monomeric globular proteins is intimately connected to destabilization of the native fold (64). Importantly, a specific intermediate of the folding pathway of wild type β_2_m, which was previously structurally characterized and shown to play a crucial role in priming the amyloid transition (47), is particularly abundantly populated by the D76N variant. It is therefore possible that this specific residue substitution facilitates the molecular mechanism responsible for the inherent amyloidogenicity of wild type β_2_m and thereby enables the variant to cause clinical pathology even at a normal plasma concentration rather than the grossly increased abundance of wild type β_2_m responsible for dialysis-related amyloidosis.

Our elucidation of the structural properties and folding dynamics of the highly amyloidogenic D76N variant has validated several earlier interpretations of the molecular basis of the amyloid transition of the wild type β_2_m. The characterization of conditions for rapid fibrillogenesis of the variant in a physiological milieu is therefore particularly significant. D76N β_2_m forms amyloid fibrils within a few hours in physiological buffers *in vitro* that is enhanced by fluid agitation and exposure to a hydrophobic surface. In contrast, fibrillogenesis of wild type β_2_m is extremely slow under physiological conditions, being minimal or absent after 100 days of incubation (61). Fluid agitation has been shown previously to be crucial in priming amyloid fibrillogenesis of other polypeptides including amyloid β (31), insulin (65), apolipoprotein C-II (66), and α-synuclein (67), but all these precursors were either natively unfolded (apolipoprotein-CII, amyloid β, and α-synuclein) or induced to unfold by a denaturing buffer (insulin).

Our present demonstration that the interfacial forces, acting in a physiologically relevant fluid flowing over natural hydrophobic surfaces, can prime fibrillar conversion of D76N β_2_m monomers identifies this protein as a genuine paradigm for amyloidogenic globular proteins causing systemic amyloidosis. Although critically destabilized by comparison with the wild type protein, it nevertheless folds in the wild type native conformation and evades intracellular quality control so that it is secreted at a physiological rate. Nevertheless, when, like all globular proteins, its stability is challenged by the physiological extracellular environment (51), the variant's propensity to misfold and aggregate as amyloid fibrils becomes evident. Within the extracellular space where amyloid is deposited in the systemic amyloidoses, the interstitial fluid flows over the extensive surfaces of the fibrous network of elastin, collagen, and leucine-rich proteoglycans (68), the high hydrophobicities of which play a key role in promoting local unfolding of globular proteins. We have previously reported the capacity of collagen to prime the formation of wild type β_2_m amyloid fibrils stacked on the collagen surface, and here we show that elastin is a potent promoter of massive amyloid conversion of the D76N variant in solution. Massive enhancement by graphite nanotubes of variant β_2_m amyloid fibrillogenesis further confirms the role of hydrophobic surfaces. Although Linse *et al.* (69) have previously noted an effect of hydrophobic surfaces on fibrillization of wild type β_2_m with accelerated nucleation induced by nanoparticles covering a range of sizes and hydrophobicity patterns, their experiments were done at the grossly non-physiological pH of 2.5.

The kinetics of fibril formation by wild type β_2_m and its truncated form ΔN6 β_2_m depend on a critical nucleation step and can be accelerated by the presence of amyloid fibril seeds. In particular, the truncated form ΔN6 β_2_m can catalyze the oligomerization of the wild type (70) and even prime the fibrillogenesis of the wild type protein in physiological buffer (61) although with a slower rate and lower yield than when primed by D76N β_2_m. The D76N variant is also much more potent than ΔN6 β_2_m in promoting formation of actual amyloid fibrils by wild type β_2_m. The apparent capacity of monomeric ΔN6 β_2_m to induce conformational rearrangement of the wild type protein structure has previously been ascribed to a prion-like effect (71). In our hands, however, monomeric D76N variant and ΔN6 β_2_m do not prime fibrillogenesis by wild type β_2_m, which only occurs when it is exposed to filaments and fibrils of the priming species. Such a mechanism is more consistent with a surface nucleation process (72) rather than a genuine prion-like effect.

The contrast between the potent *in vitro* priming and enhancement by D76N β_2_m of amyloid fibril formation by wild type β_2_m and the proteomic evidence that the wild type protein is not present in the *in vivo* amyloid deposits are intriguing, especially as wild type β_2_m clearly does form amyloid *in vivo* in dialysis-related amyloidosis. Furthermore, in other types of hereditary systemic amyloidosis in which the wild type precursor protein is mildly amyloidogenic, for example transthyretin, most patients are heterozygotes for the causative mutation, expressing both amyloidogenic variant and wild type, and both proteins are present in the amyloid fibrils. However, as we have shown here, the capacity of D76N β_2_m to catalyze fibrillogenesis by wild type β_2_m can be modulated and even blocked by typical chaperones such as crystallin, and this inhibition depends on the stoichiometric chaperone/β_2_m ratio. A role for extracellular chaperone-like proteins in the inhibition of wild type β_2_m amyloidogenesis has been proposed previously (73), and it is plausible that the persistent, extremely high concentration of wild type β_2_m in renal failure patients on dialysis may overcome the natural protective role of physiological chaperones that otherwise protect against deposition of this rather weakly amyloidogenic protein when it circulates at its normal serum concentration. In addition to illuminating the critically important biophysical features of the physiological milieu where amyloid formation takes place, our results thus open up novel avenues for exploration of hitherto unanswered questions about amyloidosis: why only a handful of all proteins ever form amyloid *in vivo*, and when, why, and where amyloid is deposited in disease.

## Footnotes

2 The abbreviations used are:

β_2_m: β_2_-microglobulin
Δ*G*: free energy change
U: unfolded state
I: intermediate
N: native state
subscript C: *cis*-His^31^-Pro^32^
subscript T: *trans*-His^31^*-*Pro^32^
TS: transition state
Δ*G*^‡^: free energy barrier
*t_m_*: mixing time for TOCSY, NOESY, and ROESY
BLUU-Tramp: Biophysics Laboratory University of Udine temperature ramp
Gdn-HCl: guanidine hydrochloride
ThT: thioflavin T
AFM: atomic force microscopy
ΔN6 β_2_m: truncated β_2_m isoform lacking the 6 N-terminal residues
TOCSY: total correlation spectroscopy
ROESY: rotating frame nuclear Overhauser enhancement spectroscopy.
